# Traumatic Brain Injury Accelerates the Onset of Cognitive Dysfunction and Aggravates Alzheimer's-Like Pathology in the Hippocampus by Altering the Phenotype of Microglia in the APP/PS1 Mouse Model

**DOI:** 10.3389/fneur.2021.666430

**Published:** 2021-09-01

**Authors:** Di Wu, Jay Prakash P. Kumal, Xiaodi Lu, Yixuan Li, Dongsheng Mao, Xudong Tang, Meitong Nie, Xin Liu, Liang Sun, Bin Liu, Yafang Zhang

**Affiliations:** ^1^Department of Neurology, Fourth Affiliated Hospital of Harbin Medical University, Harbin, China; ^2^Department of Human Anatomy, Harbin Medical University, Harbin, China; ^3^Key Laboratory of Preservation of Human Genetic Resources and Disease Control in China (Harbin Medical University), Ministry of Education, Harbin, China; ^4^Department of Anesthesiology, Harbin Medical University Cancer Hospital, Harbin, China; ^5^Department of Neurology, General Hospital of Northern Theater Command, Shenyang, China

**Keywords:** traumatic brain injury, Alzheimer's disease, Aβ plaque pathology, neuroinflammation, microglia

## Abstract

An increasing number of studies have suggested that traumatic brain injury (TBI) is associated with some neurodegenerative diseases, including Alzheimer's disease (AD). Various aspects of the mechanism of TBI-induced AD have been elucidated. However, there are also studies opposing the view that TBI is one of the causes of AD. In the present study, we demonstrated that TBI exacerbated the disruption of hippocampal-dependent learning and memory, worsened the reductions in neuronal cell density and synapse formation, and aggravated the deposition of Aβ plaques in the hippocampi of APP/PS1 mice. We also found that TBI rapidly activated microglia in the central nervous system (CNS) and that this effect lasted for at least for 3 weeks. Furthermore, TBI boosted Aβ-related microglia-mediated neuroinflammation in the hippocampi of APP/PS1 mice and the transformation of microglia toward the proinflammatory phenotype. Therefore, our experiments suggest that TBI accelerates the onset of cognitive dysfunction and Alzheimer-like pathology in the APP/PS1 mouse model, at least partly by altering microglial reactions and polarization.

## Introduction

Traumatic brain injury (TBI) is one of the leading causes of disability and death worldwide, with ~69 million people experiencing TBI every year (1). In addition to experiencing physical disabilities, TBI patients often exhibit cognitive deficits, which in turn imposes a tremendous burden on patients and their families. Furthermore, a number of neurodegenerative diseases, such as Alzheimer's disease (AD) (2–4) and chronic traumatic encephalopathy (2), occur secondary to TBI in the chronic stage.

AD is one of the most common causes of dementia. According to data from the Alzheimer's Association, by 2050, the number of patients who progress to AD will increase threefold relative to that in 2016 (5, 6). The pathology of AD is characterized by senile plaques (SPs), deposition of amyloid-β (Aβ) protein, and neurofibrillary tangles (NFTs), which are tau protein aggregates, in the brain. Despite the numerous efforts that have been made to identify the pathogenesis of AD, the causes of this disease, which may be a combination of senescence in the brain and genetic, environmental, and lifestyle factors, remain poorly understood. Epidemiological and pathophysiological studies have indicated a relationship between TBI and AD. From an epidemiological perspective, Plassman et al. showed that the rate of onset of AD is related to the severity of TBI (7). A population-based study suggested that people who experience TBI earlier than age 65 have an earlier onset age of AD than people who do not (8). TBI with loss of consciousness may also reduce the time to the onset of AD (9). From a pathophysiological perspective, the link between TBI and AD is related to the deposition of Aβ and tau protein, apolipoprotein E (ApoE) ε4 allele, dysfunction of the blood-brain barrier and neuroinflammation (10, 11), cerebral vascular factors (12), and so on. However, the topic is controversial, as some studies have argued against the idea of that TBI is a risk factor associated with AD (13, 14).

Microglia-mediated neuroinflammation is a well-elucidated pathogenesis in both TBI and AD. As central nervous system (CNS)-resident macrophages, microglia contribute to monitoring and sustaining CNS homeostasis when in the resting state. In response to harmful stimuli, such as neurotrauma, pathogens or abnormal folded proteins, microglia can be activated toward a classical (M1) phenotype to release of proinflammatory cytokines or an alternative (M2) phenotype to resolve inflammation, remove damage, and repair tissue (15). Microglia become activated within a few minutes after TBI to respond to damage and remain reactive for a few weeks to months (16). In AD, activated microglia in the M1-like state bind to abnormally deposited Aβ, produce inflammatory factors, and prevent clearance of Aβ (17–20). However, other studies have demonstrated that M2-like microglia can facilitate the phagocytosis of Aβ and recovery from neurodegenerative diseases, including AD (21, 22). Nevertheless, whether microglial activation is involved in the pathological progression of AD in the context of TBI remains to be determined.

Therefore, in this study, the effect of TBI on the exacerbation of Alzheimer's-like cognitive dysfunction and pathology in amyloid-β precursor protein (APP)/presenilin 1 (PS1) transgenic mice, as well as the potential mechanism related to the activation and phenotype of microglia following TBI, was assessed in an attempt to provide preclinical evidence supporting TBI-triggered AD progression.

## Experimental Procedures

### Animals

All animal experiments were carried out in accordance with the National Institute of Health Guide for the Care and Use of Laboratory Animals (NIH Publications No. 80–23), revised in 1996, and associated guidelines and were approved by the Ethical Committee of Harbin Medical University. A double-transgenic AD mouse model (APPswe, PSEN1dE9; APP/PS1 mice) and C57BL/6 mice, purchased from the Model Animal Research Center of Nanjing University, were housed in polypropylene cages in a standard environment on a 12-h light–dark cycle and provided free access to food and water. For all experimental procedures, 6-month-old male C57BL/6 mice weighing 25–30 g were selected and randomly divided into two groups: the C57BL/6 control group and C57BL/6 TBI group. Six-month-old male APP/PS1 mice weighing 25–30 g were selected and randomly divided into two groups: the APP/PS1 control group and APP/PS1 TBI group.

### Experiment Design

In the current study, mild TBI (mTBI) was induced in mice. On the 29^th^ and 30^th^ days post-mTBI, the novel object recognition (NOR) test and object place recognition (OPR) test were performed to test learning and memory function. After the cognitive function tests, all mice were sacrificed, and the brain tissues were dissected. To determine whether Alzheimer's-like pathology and synaptic impairments can be accelerated by mTBI at a relatively early chronic stage (23, 24), HE staining, immunofluorescence (IF) staining, and immunohistochemistry (IHC) were performed to evaluate cell density in the brain, the degree of Aβ plaque deposition in the brain, and SYN expression in the brain. Furthermore, the activation and phenotype of microglia were determined by IF staining and Western blotting after the 1st, 2nd, 3rd, and 4th weeks of TBI.

### Protocols of mTBI

In the TBI group, mTBI was induced by the weight drop method as previously described (25, 26). Mice were anesthetized using 10% chloral hydrate, and an incision was made in the scalp along the sagittal suture to expose the hypodermis with vessel forceps. The periosteum was stripped off the right parietal bone with a bistoury, and a 5-mm-diameter hole was made with a bone drill to expose the endocranium. To cause cortical injury, a hammer (10 g) was released from a height of 3 cm above the head so that it struck the rod connected to the dura. In the control groups, the mice underwent all surgical procedures except cortical impact.

### Hematoxylin-Eosin Staining

HE staining was performed on 3-μm-thick paraffin-embedded brain tissue sections. Sections were deparaffinized, hydrated in descending concentrations of ethanol, and stained with hematoxylin and eosin. The sections were washed with PBS, dehydrated in ascending concentrations of ethanol, and mounted with standard neutral balsam. The sections were photographed using a microscope, and the cell density was measured using Adobe Photoshop CC 2018.

### NOR Test

The NOR test was performed in a blue plastic box (35 × 35 × 20 cm^3^). The test was performed without habituation to the empty field as described previously (27, 28). In the acquisition phase, the mice were exposed to two identical plastic objects that were placed in two contiguous corners for 10 min. During the test phase, which took place 1 h later, one of the objects was replaced with a novel object in the same position, and each mouse was allowed to explore the field and objects for 5 min. The box and objects were thoroughly cleaned with 75% ethanol between the trials to eliminate olfactory cues. Exploration was defined as the head of the animal facing the object at a distance <1 cm or physically touching the object with the upper limbs. The time spent exploring the objects during the acquisition and testing phase was recorded for each mouse. The preference index is expressed as the percentage of time spent exploring the novel object related to the total exploration time.

### OPR Test

The OPR test (29) was performed in the same apparatus without habituation to the empty field. The acquisition phase was the same as that in the NOR test. In the test phase, which took place 1 h later, one of the two objects was moved to the diagonal corner, and each mouse was allowed to explore the field and objects for 5 min. The box and objects were thoroughly cleaned with 75% ethanol between the trials to eliminate olfactory cues. The time spent exploring the objects during the acquisition and testing phase was recorded for each mouse. The definitions of exploration and preference index were the same as in the NOR test.

### IHC Staining

IHC staining was performed on 3-μm-thick paraffin-embedded brain tissue sections. The sections were deparaffined and boiled in citric acid buffer solution for 5 min. After they were cooled to room temperature, the sections were incubated with an endogenous peroxidase blocking solution (catalog number: PV-6002, ZSGB-BIO, Beijing, China) for 15 min and then blocked with serum. To detect Aβ plaque deposits, the sections were incubated with a mouse anti-Aβ primary antibody (1:100; catalog number: #15126S; Cell Signaling Technology, Cambridge, MA, USA) overnight. Next day, the sections were washed with PBS and incubated with a horseradish peroxidase (HRP)-conjugated goat anti-mouse IgG (H+L) secondary antibody (catalog number: PV-6002; ZSGB-BIO, Beijing, China). The sections were incubated with dimethylaminobenzaldehyde (DAB) reagent (Beyotime Biotechnology, Zhejiang, China) to induce a chromogenic reaction. The sections were counterstained with hematoxylin and mounted with a coverslip.

### IF Staining

IF staining was performed on 3-μm-thick paraffin-embedded mouse brain sections. The sections were deparaffined and boiled in citric acid buffer solution for 5 min. After being cooled to room temperature, the sections were permeabilized in 1% Triton X-100 in PBS for 5 min. Then, the sections were blocked with serum. To detect *in situ* antigens, the sections were incubated with primary antibodies [mouse anti-Aβ (1:100; catalog number: #15126S; Cell Signaling Technology), rabbit anti-synaptophysin (SYN) (1:100, catalog number: 17785-1-AP; Proteintech, Chicago, IL, USA), and rabbit anti-ionized calcium-binding adapter molecule 1 (IBA-1) (1:100, catalog number: 10904-1-AP; Proteintech)] overnight. Successively, the sections were washed by PBS and incubated with secondary antibodies [Alexa Fluor 594-conjugated goat anti-rabbit IgG (H+L) cross-adsorbed ReadyProbes secondary antibody (catalog number: #R37117) and Alexa Fluor 488-conjugated goat anti-mouse IgG (H+L) highly cross-adsorbed secondary antibody (catalog number: #A-11034) (1:2,000, Invitrogen; Thermo Fisher Scientific, Carlsbad, CA, USA)]. The sections were counterstained with 4′,6-diamidino-2-phenylindole (DAPI) (1 μg/ml; Sigma-Aldrich) and mounted with coverslips, and photos were captured under a BX51 fluorescence microscope (Olympus, Tokyo, Japan).

### Western Blotting

The hippocampus was dissected out from APP/PS1 and APP/PS1 TBI mice. Western blotting was performed as described previously (30). Target protein expression was measured using the following primary antibodies: rabbit anti-arginase-1 (Arg1) (catalog number: #93668; Cell Signaling Technology), rabbit anti-inducible nitric oxide synthase (iNOS) (catalog number: #13120; Cell Signaling Technology), and rabbit anti-β-actin (catalog number: #4790; Cell Signaling Technology) (IgGs; 1:2000). β-Actin was utilized as an internal reference. The protein blots were visualized using an HRP-conjugated goat anti-rabbit IgG (H + L) secondary antibody (1:5000, Invitrogen; Thermo Fisher Scientific) and a Chemi-Doc™ Imaging System (Bio-Rad Laboratories, Inc., Hercules, CA, USA) and analyzed using Image-Pro Plus 6.0.

### Morphometric Analysis

Mouse brains were serially sectioned according to the mouse brain atlas. For HE staining, IF staining, and IHC, adjacent serial sections were used. For quantitative analyses of neuronal cell density and the integrated optical density (IOD) of specific markers in the cortex and hippocampus, three fields per slide containing the cortex, CA1, CA2, CA3, and dentate gyrus (DG) regions were evaluated. The neuronal cell density was calculated using the Photoshop software by counting only neuronal nuclei with a typical morphology. IF staining and IHC were performed using Image-Pro Plus 6.0. Pictures were imported into the software and converted to grayscale. For IF staining, the IOD count range was set as follows: 20–255 for IBA-1, 35–255 for SYN, and 50–255 for Aβ. For IHC for Aβ, the IOD count range was set from 0 to 100.

### Statistical Analysis

The data are expressed as the mean ± standard error of the mean (SEM). The data were analyzed by one-way analysis of variance (ANOVA) followed by Bonferroni's *post-hoc* multiple comparisons test. All statistical analyses were carried out using GraphPad Prism 7.0 software. A *p* < 0.05 was considered statistically significant.

## Results

### The Cognitive Function of APP/PS1 Mice Deteriorated After TBI

**Figure 1:**
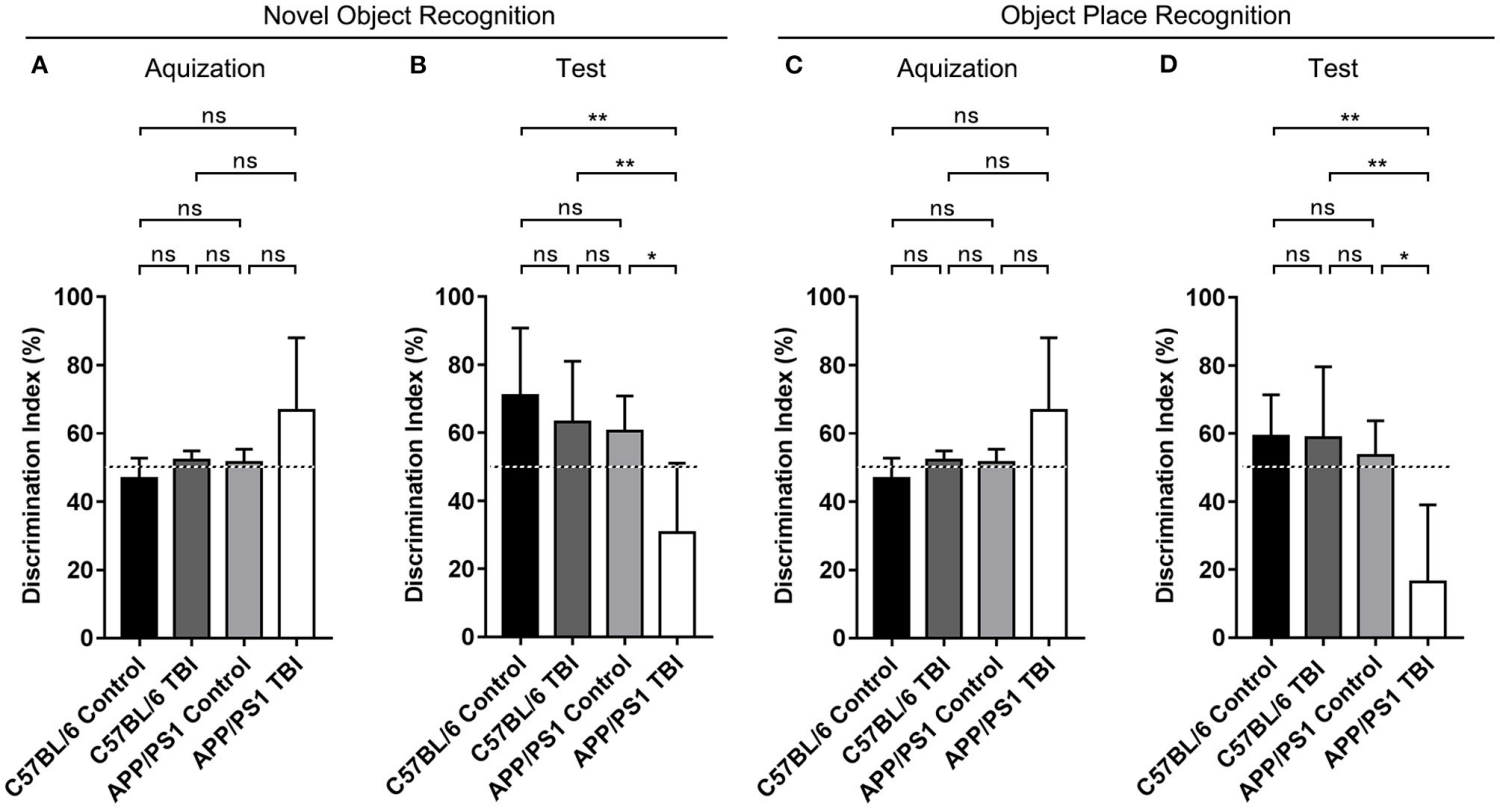
The cognitive function of APP/PS1 mice deteriorated after TBI. Quantitative analyses of the discrimination indexes of C57BL/6, C57BL/6 TBI, APP/PS1, and APP/PS1 TBI group mice in the acquisition **(A)** and test phases **(B)** in the NOR test (*n* = 8/group). Quantitative analyses of the discrimination indexes of C57BL/6, C57BL/6 TBI, APP/PS1, and APP/PS1 TBI group mice in the acquisition **(C)** and test phases **(D)** in the OPR test (*n* = 8/group). ns, non-significant; ^*^p < 0.05; ^**^p < 0.01.

We generated double-transgenic AD (APP/PS1) and C57BL/6 mouse models of mTBI and performed the NOR and OPR tests to assess the cognitive function of each group 4 weeks after mTBI. The results showed that during the acquisition phase, each group of mice spent almost the same amount of time exploring the two identical objects (*p* > 0.05) (Figures 1A,C). During the test phase 1 h later, the mice in the C57BL/6, C57BL/6 TBI, and APP/PS1 groups preferred to spend more time exploring the novel object and the object in a novel place and showed no between-group differences, excluding the possibility that mTBI alone had a significant negative effect on hippocampus-dependent cognition [C57BL/6: *p* > 0.05 vs. C57BL/6 TBI; C57BL/6: *p* > 0.05 vs. APP/PS1; C57BL/6 TBI: *p* > 0.05 vs. APP/PS1; C57BL/6, C57BL/6 TBI and APP/PS1: *p* < 0.05 vs. chance (50%)] (Figures 1B,D). However, compared to uninjured AD mice, APP/PS1 TBI mice showed no preference for the novel object or the displaced object (APP/PS1 TBI: *p* < 0.05 vs. APP/PS1). These results indicated that TBI exacerbated the impairment of hippocampal-dependent learning and memory in APP/PS1 mice.

### Cell Density in the CA3 Region of the Hippocampus in APP/PS1 Mice Decreased After TBI

**Figure 2:**
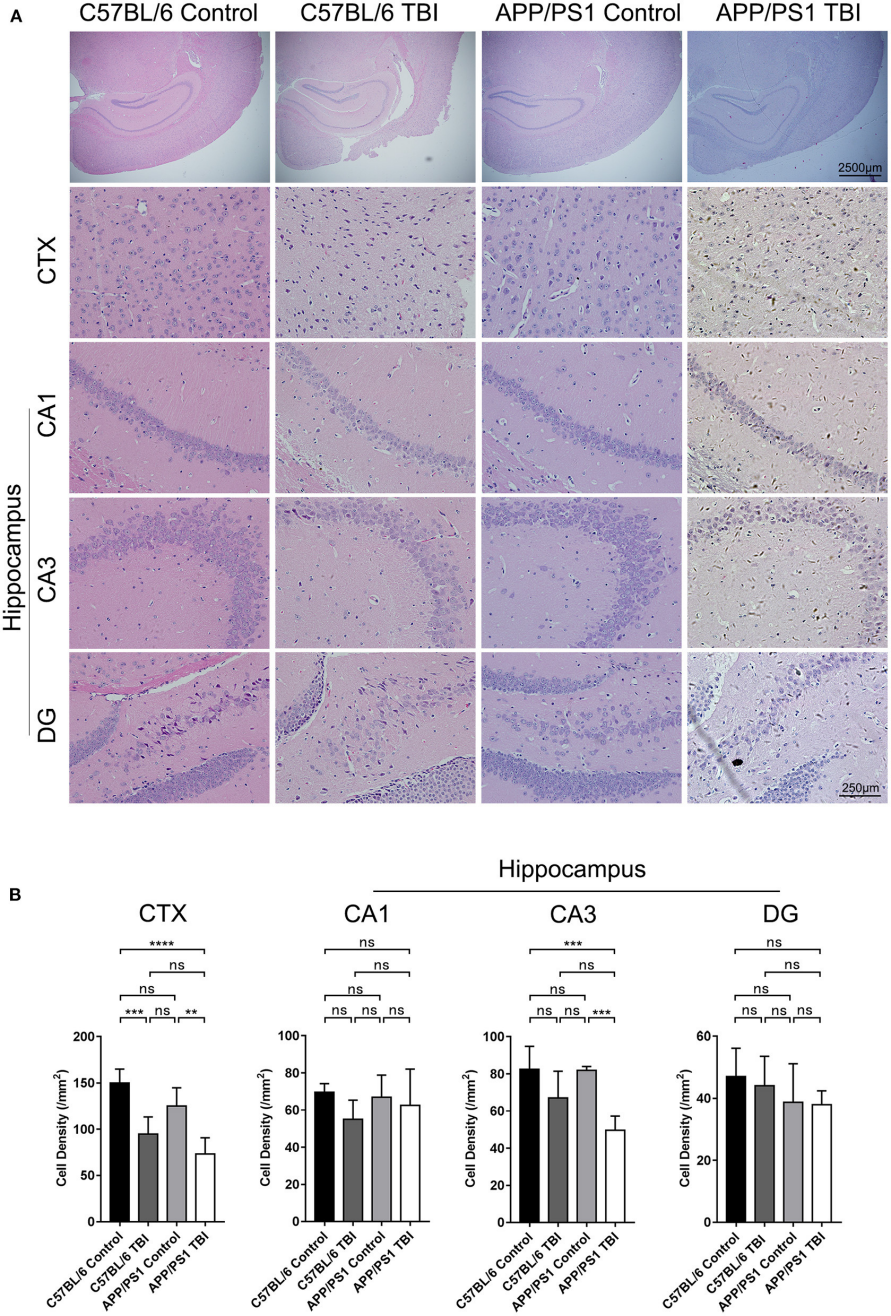
Cell density in the CA3 region of the hippocampus in APP/PS1 mice decreased after TBI. **(A)** Photographs of HE staining of cortical and hippocampal (CA1, CA3, and DG) tissues from C57BL/6, C57BL/6 TBI, APP/PS1, and APP/PS1 TBI group mice. **(B)** Cell density in the cortex and hippocampus (CA1, CA3, and DG) in C57BL/6, C57BL/6 TBI, APP/PS1, and APP/PS1 TBI group mice. The error bars represent the SEM (*n* = 8/group). ns, non-significant; ^**^p < 0.01; ^***^p < 0.001.

TBI was induced in the brain cortex, and a decrease in cortical cell density was observed (C57BL/6 TBI: *p* < 0.001 vs. C57BL/6; APP/PS1 TBI: *p* < 0.01 vs. APP/PS1) (Figure 2). However, the hippocampus was not injured directly. It is well-known that the hippocampus is an important area related to cognition, memory, and emotion (31). As shown by HE staining, neurons in the hippocampus, which were identified according to the typical morphology of the nuclei, were obviously disorganized in mice subjected to TBI. Our results further revealed that the cell density of neurons in the CA3 region was similar between C57BL/6 mice and APP/PS1 mice (APP/PS1: *p* > 0.05 vs. C57BL/6) (Figure 2). After TBI, there was a decreasing trend, although not significant, in cell density in C57BL/6 TBI group mice compared to C57BL/6 group mice (C57BL/6 TBIL *p* = 0.18 vs. C57BL/6) (Figure 2). Notably, the cell density of neurons in the CA3 area in APP/PS1 TBI mice was significantly lower than that in APP/PS1 mice (APP/PS1 TBI: *p* < 0.001 vs. APP/PS1) (Figure 2). Regarding the cell density in the other regions of the hippocampus, there was no significant difference between the groups (Figure 2). These results indicated that TBI led to a decrease in the neuronal density in the CA3 region in APP/PS1 mice, which might have to some extent accounted for the post-TBI cognitive impairment in AD model mice.

### Synapse Formation in the Cortex and Hippocampus Deteriorated in APP/PS1 Mice After TBI

**Figure 3:**
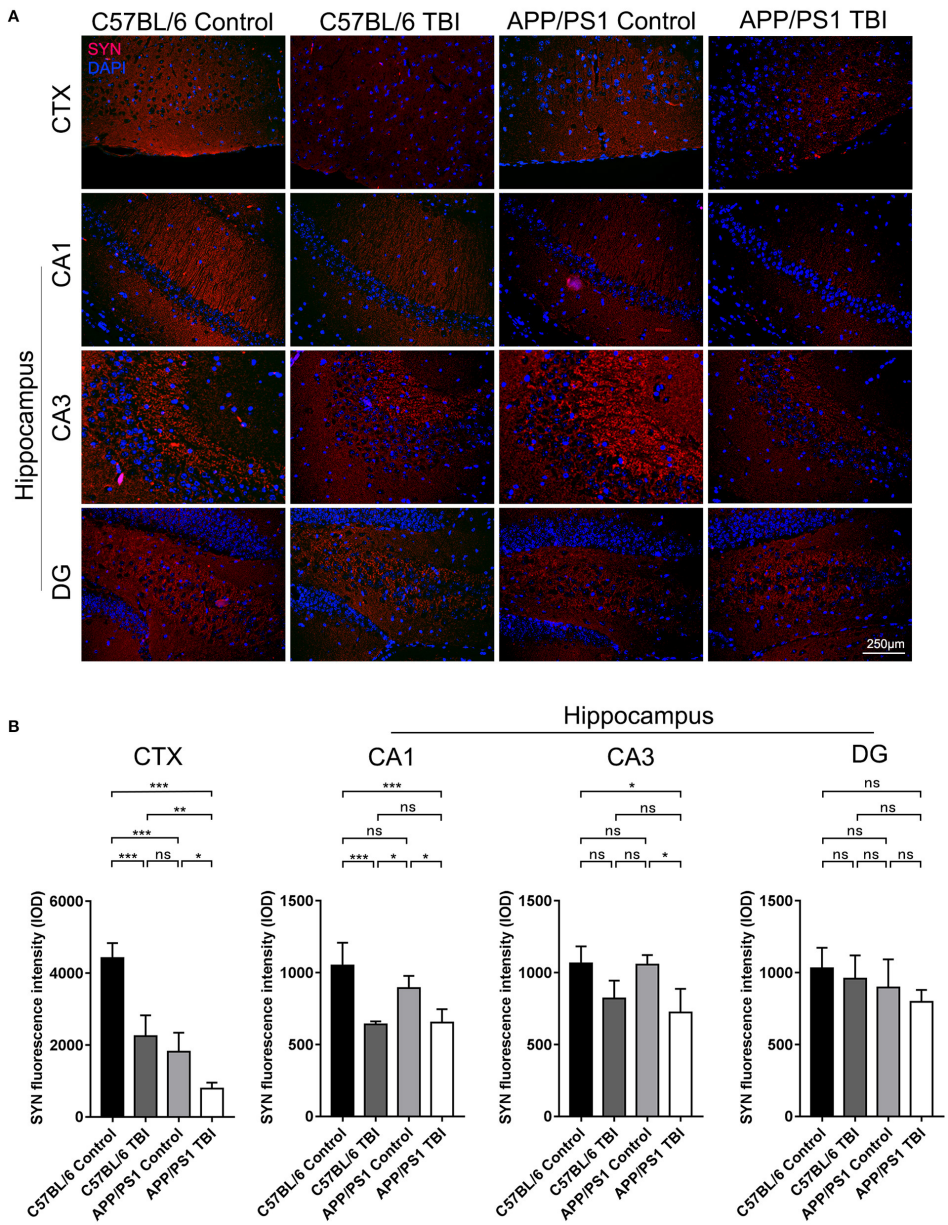
Synapse formation in the cortex and hippocampus deteriorated in APP/PS1 mice after TBI. **(A)** Representative fluorescence images of cortical and hippocampal (CA1, CA3, and DG) tissues from in C57BL/6, C57BL/6 TBI, APP/PS1, and APP/PS1 TBI group mice immunostained for SYN (in red) and subjected to nuclear staining with DAPI (in blue). **(B)** The fluorescence intensity of SYN in the cortex and in the hippocampus (CA1, CA3, and DG). The error bars represent the SEM (*n* = 8/group). ns, non-significant; ^*^p < 0.05; ^**^p < 0.01; ^***^p < 0.001.

SYN is a canonical marker of synapses that indicates synaptogenesis (32). To further evaluate synapse formation in the brain in each group, IF staining of SYN was performed to evaluate its expression in the cortex and hippocampus. C57BL/6 mice exhibited abundant SYN expression in the cortex. SYN expression was lower in APP/PS1 mice than C57BL/6 mice (APP/PS1 TBI: *p* < 0.001 vs. APP/PS1), indicating impaired synaptogenesis in AD model mice (Figure 3). As expected, the expression of SYN in the cortex in APP/PS1 TBI group mice was significantly decreased compared with that in the APP/PS1 group mice (APP/PS1 TBI: *p* < 0.05 vs. APP/PS1), which was indicative of further disruption of synapse formation and in parallel with cognitive decline (Figure 3). Similarly, in the CA1 and CA3 regions of the hippocampus, the expression of SYN in APP/PS1 TBI group mice was also significantly decreased compared to that in APP/PS1 group mice (APP/PS1 TBI: *p* < 0.05 vs. APP/PS1). These results suggested that synaptic structure was further deteriorated in the AD mouse model after TBI.

### TBI Led to Long-Term Activation of Microglia in the Brain

**Figure 4:**
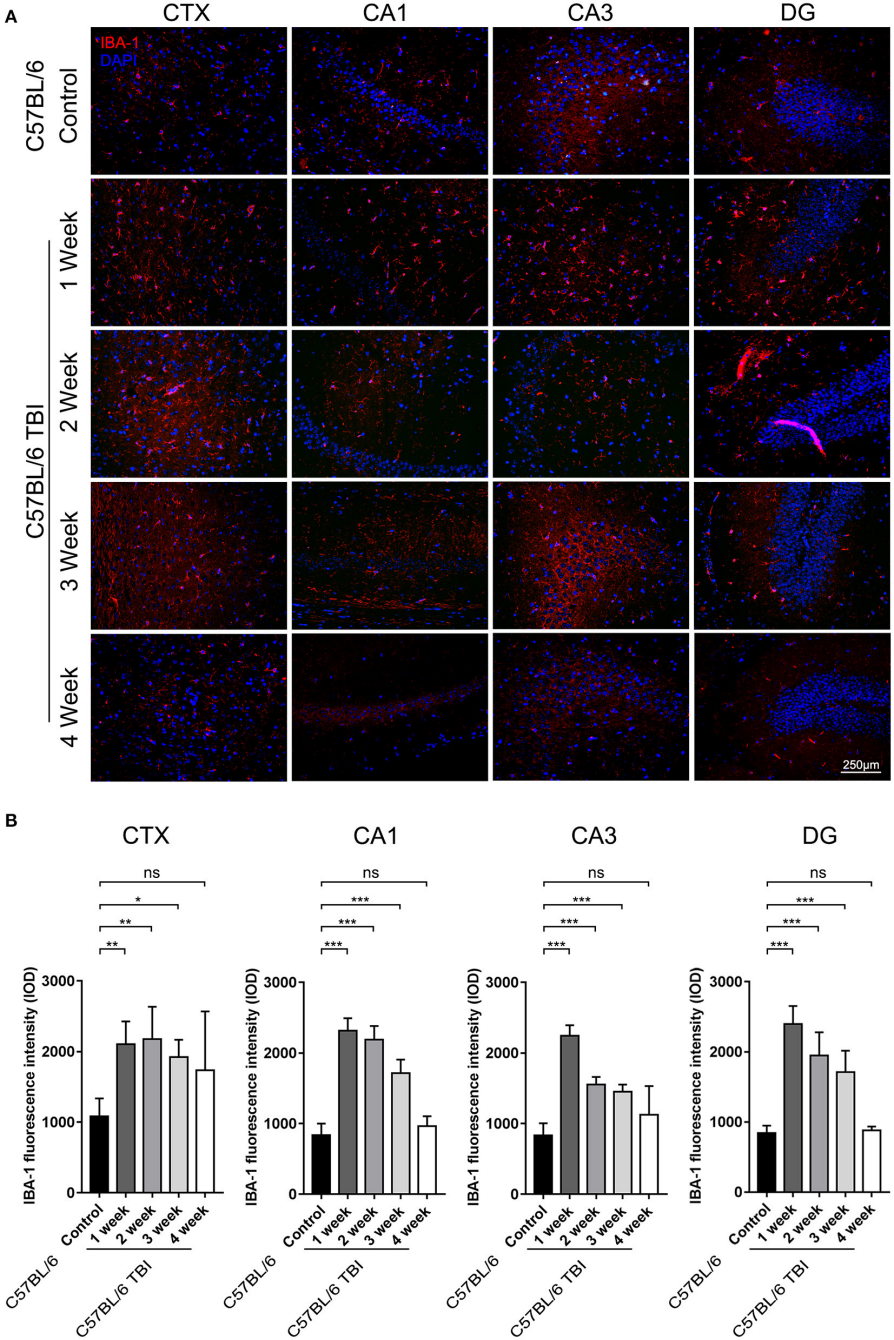
TBI led to long-term activation of microglia in the brain. **(A)** Representative fluorescence images of cortical and hippocampal (CA1, CA3, and DG) tissues from C57BL/6 and C57BL/6 TBI group mice immunostained for IBA-1 (in red) and subjected to nuclear staining with DAPI (in blue). **(B)** The fluorescence intensity of IBA-1 in the cortex and in the hippocampus (CA1, CA3, and DG). The error bars represent the SEM (*n* = 8/group). ns, non-significant; ^*^p < 0.05; ^**^p < 0.01; ^***^p < 0.001.

Microglia can be quickly activated within hours to days after TBI (33). To further determine the duration of post-TBI microglial activation, we performed IF staining to detect IBA-1-positive cells in the first, second, third, and fourth weeks after TBI. To avoid detecting the changes caused by Aβ plaque deposition, which may also increase microglial activation, continuous monitoring of the expression of microglial markers was performed only in C57BL/6 mice. The results showed that the number of IBA-1^+^ microglia was significantly increased in the cortex and hippocampus after the 1st week after TBI (Figure 4) and that microglial activation lasted for at least 3 weeks (C57BL/6: *p* < 0.05 vs. C57BL/6 TBI in the 1st week; C57BL/6: *p* < 0.01 vs. C57BL/6 TBI in the 2nd week; C57BL/6: *p* < 0.01 vs. C57BL/6 TBI in the 3rd week; C57BL/6: *p* > 0.05 vs. C57BL/6 TBI in the 4th week) (Figure 4).

### TBI Aggravated Aβ Plaque Deposition in the Hippocampus in the APP/PS1 Mouse Model

**Figure 5:**
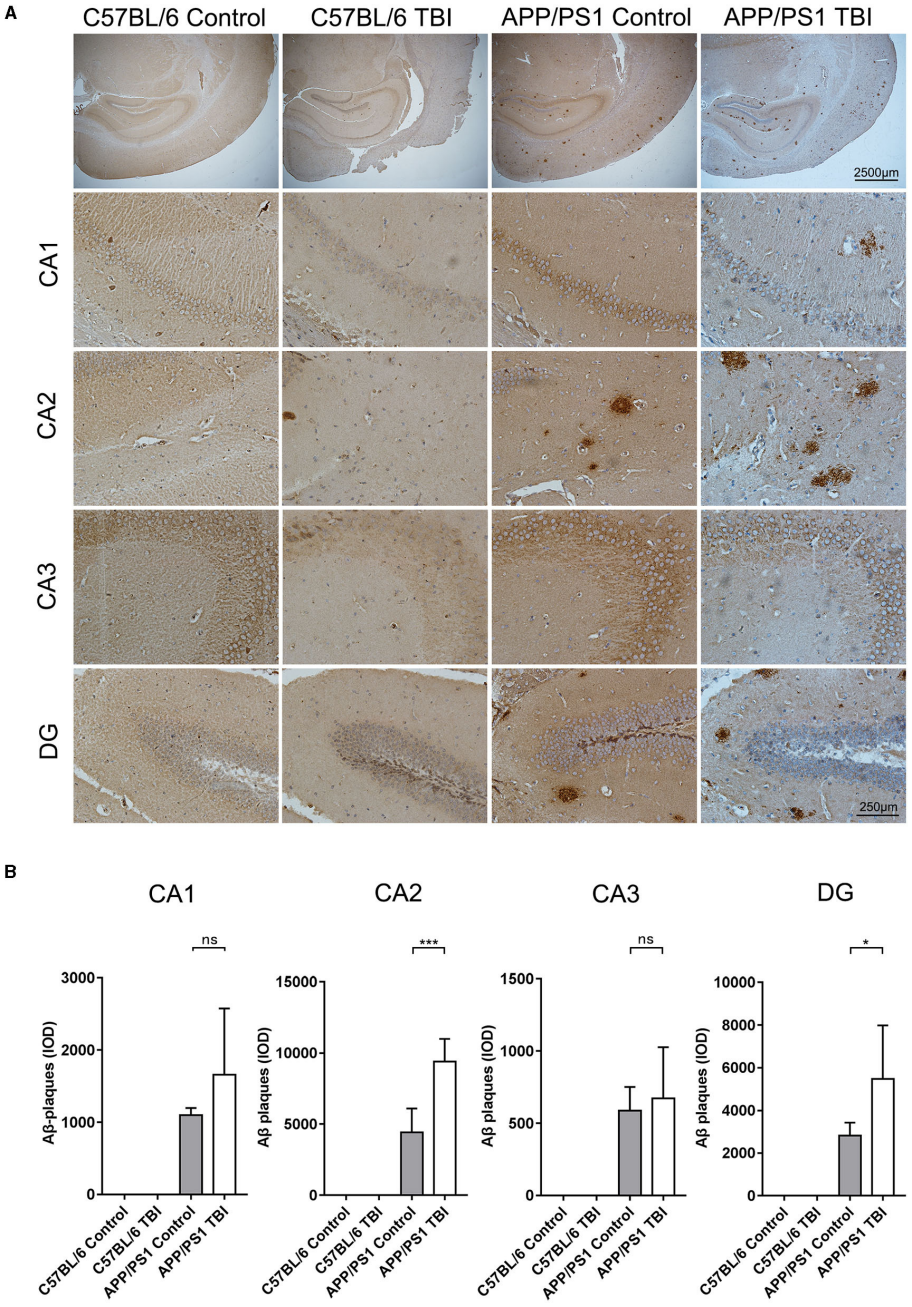
TBI aggravated Aβ plaque deposition in the hippocampus in the APP/PS1 mouse model. **(A)** Representative IHC images of hippocampal (CA1, CA2, CA3, and DG) tissues from C57BL/6, C57BL/6 TBI, APP/PS1, and APP/PS1 TBI group mice immunostained for Aβ plaques. **(B)** Quantitative analyses of Aβ plaque deposition in the CA1, CA2, CA3, and DG regions of the hippocampus in C57BL/6, C57BL/6 TBI, APP/PS1, and APP/PS1 TBI mice. The error bars represent the SEM (*n* = 8/group). ns, nonsignificant; ^*^p < 0.05; ^***^p < 0.001.

Aβ plaque deposition is the most distinctive and classical pathological change in the AD brain. Generated by cleavage of APP by β-secretase, Aβ molecules can aggregate to form oligomers and fibrils, both of which are toxic to the CNS. As previously reported, upregulated mRNA expression of APP and increased activity of β-secretase, resulting in an abundance of materials for the generation of Aβ plaques via β-secretase, has been observed *in situ* in a TBI mouse model (34, 35). IHC was performed to further investigate whether TBI can aggravate Aβ plaque deposition in the hippocampus in APP/PS1 mice. As expected, there were no Aβ plaque deposits in C57BL/6 and C57BL/6 TBI group mice. IHC revealed that Aβ plaque deposits were mostly present in hippocampal regions (Figure 5). Further quantitative analysis revealed only a few Aβ plaques in the brains of uninjured APP/PS1 mice. However, 4 weeks after TBI, the APP/PS1 TBI group mice had significantly more Aβ plaque deposits in the hippocampus than age-matched APP/PS1 mice not subjected to TBI (APP/PS1 TBI: *p* < 0.05 vs. APP/PS1) (Figure 5), which indicated that TBI could trigger the acceleration of hippocampal Aβ plaque deposition.

### Aβ Plaque-Related Microglia-Mediated Neuroinflammation in the Hippocampi of APP/PS1 Mice Was Aggravated Post-TBI

**Figure 6:**
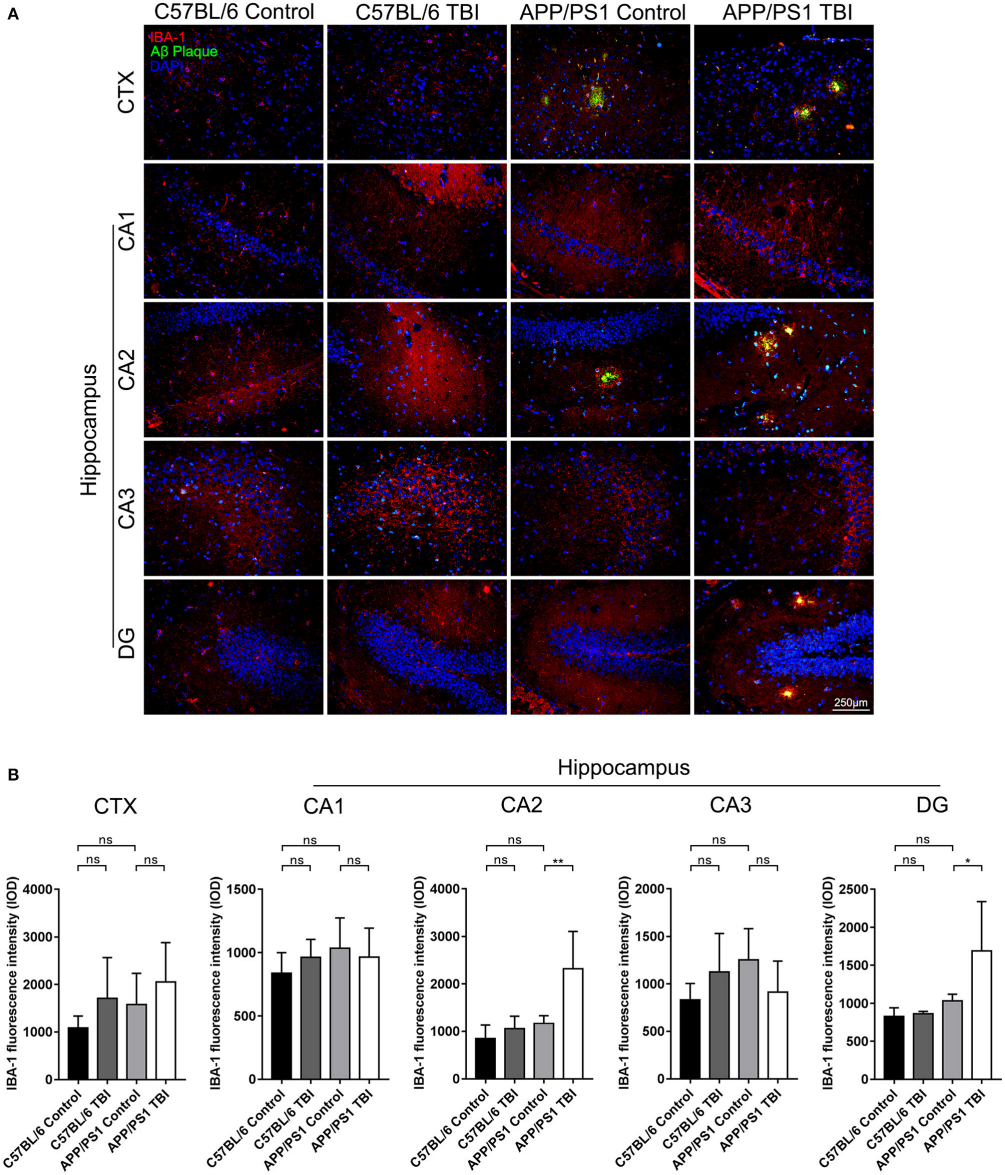
Aβ plaque-related microglia-mediated neuroinflammation in the hippocampi of APP/PS1 mice was aggravated post-TBI. **(A)** Representative fluorescence images of cortical and hippocampal (CA1, CA2, CA3, and DG) tissues from C57BL/6, C57BL/6 TBI, APP/PS1, and APP/PS1 TBI group mice immunostained for IBA-1 (in red) and for Aβ plaques (in green) and subjected to nuclear staining with DAPI (in blue). **(B)** The fluorescence intensity of IBA-1 in the cortex and in the hippocampus (CA1, CA2, CA3, and DG). The error bars represent the SEM (*n* = 8/group). ns, non-significant; ^*^p < 0.05; ^**^p < 0.01.

Microglia play an important role in the progression of AD. They can be activated by the deposition of Aβ plaques, and potent activation of microglia results in the persistent release of proinflammatory cytokines, which cause secondary injury to the CNS (36). To further determine the effects of TBI on the Aβ plaque-related activation of microglia in the hippocampus, IF double staining was performed to detect IBA-1^+^ microglia surrounding Aβ plaques. IBA-1^+^ microglia were observed around Aβ plaques in cortical and hippocampal areas in uninjured APP/PS1 mice, but there was no difference in the total number of microglia in these regions between uninjured the APP/PS1 group and C57BL/6 group (APP/PS1: *p* > 0.05 vs. C57BL/6). However, in parallel with the accelerated Aβ plaque pathology, the activation and recruitment of Aβ plaque-related microglia were significantly enhanced in the CA2 and DG regions in APP/PS1 TBI group mice compared with APP/PS1 group mice (APP/PS1 TBI: *p* < 0.01 vs. APP/PS1 in the CA2 region; APP/PS1 TBI: *p* < 0.05 vs. APP/PS1 in the DG region) (Figure 6), suggesting that Aβ plaque-related microglia-mediated neuroinflammation was aggravated in the hippocampus post-TBI.

### TBI Aggravated Alzheimer's-Like Pathology by Altering the Phenotype of Microglia

**Figure 7:**
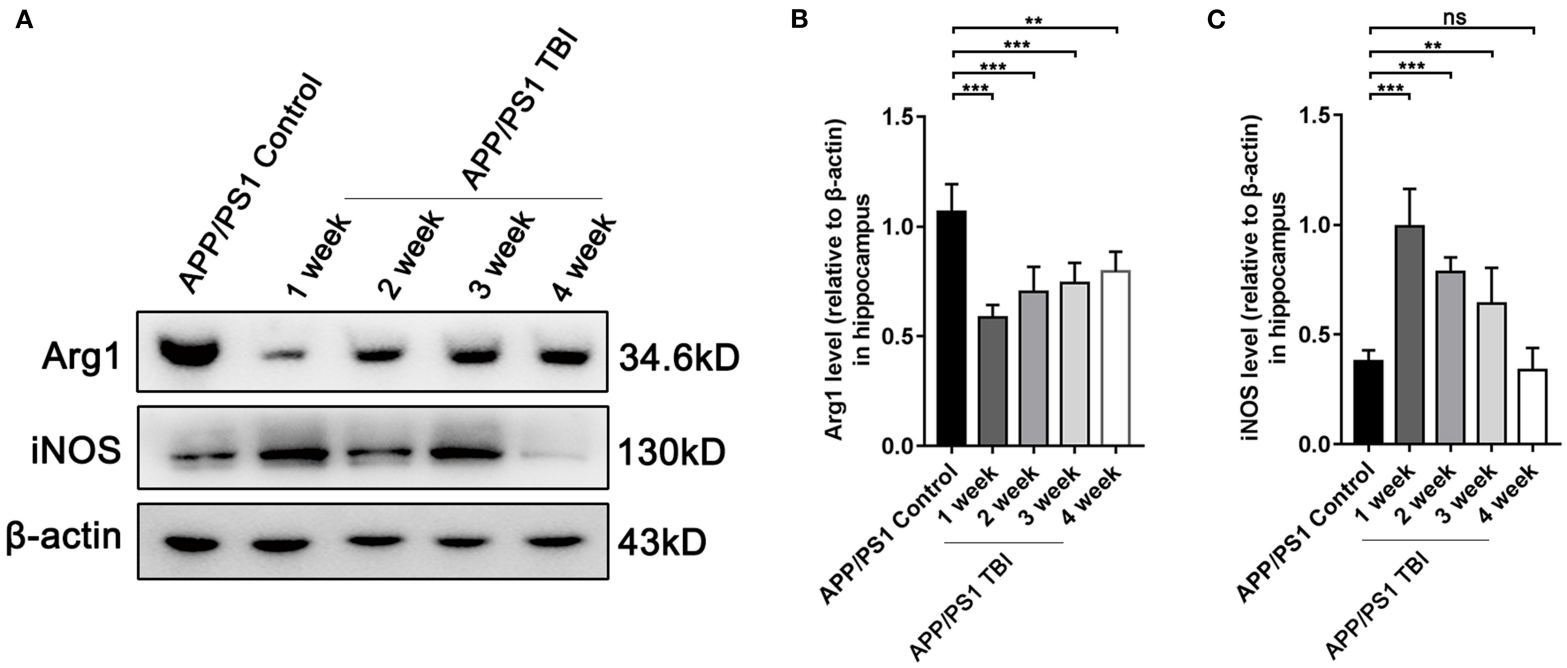
TBI aggravated Alzheimer's-like pathology by altering the phenotype of microglia. **(A)** Representative Western blotting images of Arg1, iNOS, and β-actin in the hippocampi of APP/PS1 and APP/PS1 TBI group mice. **(B,C)** Quantitative analyses of Arg1 **(B)** and iNOS **(C)** levels relative to β-actin expression in the hippocampi of APP/PS1 and APP/PS1 TBI group mice. The error bars represent SEM (*n* = 8/group). ns, non-significant; ^**^p < 0.01; ^***^p < 0.001.

As elucidated above, microglia were activated after TBI in hippocampal tissues affected by Alzheimer's-like pathology. However, the microglial phenotype that predominates in the damaged brain in the context of AD deserves further study. To further investigate the polarization of microglia, we performed Western blotting of tissues from APP/PS1 subjected to TBI or not to assess the responses of microglia. The results showed that under uninjured conditions, microglia were polarized toward the alternative (M2) phenotype and produced a high level of Arg1 (Figures 7A,B). However, microglia transitioned to the classical (M1) phenotype and produced more iNOS post-TBI (Figures 7A,C). These results indicated that microglia switched their phenotypes from an “M2-like” phenotype to an “M1-like” phenotype, a phenotype that is not beneficial for the reversal of Alzheimer's-like pathology, after TBI.

## Discussion

The mechanisms by which TBI triggers neurodegenerative diseases such as AD remain elusive. In the current study, we showed that TBI exacerbated the impairment of hippocampal-dependent learning and memory, the decrease in cell density in the hippocampus, and the loss of synapses in the brain and aggravated the accumulation of Aβ plaques in the hippocampus and Aβ-related CNS neuroinflammation in APP/PS1 mice. In summary, our data support the view that Alzheimer's-like pathology and cognitive dysfunction can be accelerated by TBI.

According to the Glasgow Coma Scale, the severity of TBI in humans can be classified into three groups: mild, moderate, and severe (10). In canonical rodent models of weight-drop-induced TBI, the severity of TBI is determined by the hammer's weight and the height from which the hammer is released (37). In the current study, mTBI, which is thought to be associated with neurodegenerative diseases and neuropsychiatric disorders in humans (38, 39), was simulated in mice by the weight-drop method as reported previously (26).

AD is a neurodegenerative disease that primarily affects cognitive function. TBI may also cause cognitive dysfunction (40). However, a single mTBI has not been reported to be associated with a high incidence of chronic cognitive impairment (41). In addition, Marschner et al. found that in the normal state, a single mTBI can indeed cause cognitive function decline that is transient and reversible (42). Moreover, it is thought that a single mTBI can result in cognitive decline in combination with peculiar conditions, such as hypertension (43), loss of consciousness (44), and perhaps AD. In our experiment, to assess whether TBI can aggravate cognitive impairment in the APP/PS1 mouse model, the NOR and OPR tests were performed to evaluate perirhinal cortex- and hippocampal-dependent learning and memory, respectively (29). Compared with C57BL/6 group mice, C57BL/6 TBI group mice showed almost no cognitive decline (Figure 1), which further excluded the possibility that mTBI alone has significant negative cognitive effects. In contrast, APP/PS1 TBI group mice exhibited less interest in the novel object and novel place (Figure 1), indicating that their short-term memory was impaired compared to that of APP/PS1 group mice. The NOR test assesses short-term working memory (45), a kind of declarative memory stored in the medial temporal lobe and hippocampus (46). It has been elucidated that spontaneous object recognition in the NOR test is more relevant to perirhinal cortex activity (47) than hippocampal function, but the OPR memory evaluated by the OPR test is considered hippocampal-dependent (29). Our data provide evidence that the onset of cognitive impairment in the APP/PS1 mouse model was dramatically accelerated after mTBI.

Synapse integrity is the basis of cognitive function. Synaptic impairment is regarded as the most common secondary pathological event downstream primarily of amyloidosis and is closely correlated with cognitive decline in AD (48, 49). In our experiments, at the age of 6 months, compared with C57BL/6 mice, APP/PS1 mice exhibited no detectable synaptic defects (Figure 3). However, compared with uninjured transgenic AD mice, APP/PS1 TBI group mice exhibited obvious failure of synapse formation (Figure 1), suggesting that mTBI facilitated synaptic pathology in the AD mouse model (Figure 3). Moreover, the earlier emergence of synaptic dysfunction might at least partly account for the accelerated onset of cognitive decline in AD model mice subjected to TBI.

The abnormal accumulation of neurotoxic Aβ is one of the well-defined fundamental hallmarks of AD pathology. The predominant molecular forms of Aβ are Aβ_1−40_ and Aβ_1−42_, which can exist as monomers, oligomers, and Aβ fibrils and can ultimately aggregate into deposits in the CNS. However, the misfolded forms of these proteins are the most cytotoxic to the CNS (50). Hajime et al. reported a transient increase in the production of Aβ post-TBI (51). Similarly, our results showed that TBI promotes the deposition of Aβ plaques in the DG and the area under the CA2 region of the hippocampus, which might disturb information input from the entorhinal cortex and transmission from the DG to the CA3 and CA1 regions (52), in APP/PS1 TBI mice (Figure 5). The progression of Aβ pathology was consistent with synaptic dysfunction in the hippocampus (Figure 4), indicating that TBI accelerated the genesis of Alzheimer's-like pathology in hippocampal regions.

Microglia-mediated neuroinflammation plays crucial roles in both AD and TBI pathogenesis. In AD, the function of microglia surrounding Aβ plaques has not yet been completely clarified. Recent studies have demonstrated that some functional molecules specifically expressed in microglia, such as trigger receptor expressed in myeloid cells 2 (TREM2), are significantly associated with the risk of AD, which suggests that microglia not merely passively respond to CNS insults but also actively contribute to the development of this disease (4).

In our experiments, mTBI led to chronic activation of cortical and hippocampal microglia that lasted for 3 weeks, which is indicative of the long-term impacts of mTBI on the microglial reaction, in C57BL/6 mice without a transgenic AD background (Figure 4). As expected, Aβ plaque-related microglial activation was marked in the CA2 and DG hippocampal regions in APP/PS1 mice in the 4th week post-TBI (Figure 6). Our data support the idea that TBI can provoke a prolonged neuroinflammatory reaction of microglia to abnormally accumulated Aβ plaques.

Activated microglia can exhibit a classical M1 polarization phenotype to release proinflammatory and neurotoxic cytokines or an alternative M2 phenotype to resolve inflammation and removal damage and repair tissue under different conditions following TBI and in AD (15, 53). However, the polarization phenotype of reactive microglia in AD model mice subjected to TBI remains elusive. Previous studies have revealed that microglia exhibit phagocytotic activity and degrade Aβ plaques (54) but that these activities are inhibited by exposure to proinflammatory cytokines and restored by anti-inflammatory chemicals (54). Additionally, M2-like polarization of microglia induced by anti-inflammatory therapy is beneficial for both cognitive function and Aβ plaque clearance in APP/PS1 mice in the early stage of AD (55). In this study, we speculated that accelerated neurodegeneration and Aβ pathology in the brain are associated with M1/M2 phenotypic switching of microglia post-TBI. Our data demonstrated that microglia in the brains of transgenic AD mice were rapidly activated and exhibited a proinflammatory phenotype after TBI that lasted for at least 3 weeks, expressing high levels of the M1 marker iNOS and decreased expression of the M2 marker Arg1 (Figure 7). These results suggested that TBI resulted in a reduction in the proportion of neuroprotective M2 microglia and an increase in the proportion of neuroinflammatory M1 microglia, leading to the accumulation of toxic Aβ and exacerbation of synaptic and cognitive dysfunction.

## Conclusion

In summary, our data provide evidence that TBI exacerbates the impairment of hippocampal-dependent learning and memory, worsens the reductions in the neuronal density and synapse formation in the hippocampus, and aggravates Aβ plaque deposition and Aβ-related microglia-mediated neuroinflammation in the hippocampus of APP/PS1 mice. TBI can exacerbate the pathology of AD and accelerate the onset of AD at least partly by altering microglial activation and phenotype in the APP/PS1 mouse model.

## Data Availability Statement

The original contributions presented in the study are included in the article/supplementary material, further inquiries can be directed to the corresponding author/s.

## Ethics Statement

The animal study was reviewed and approved by ethical committee of Harbin Medical University.

## Author Contributions

LS, BL, and YZ conceived the study. JK, XDL, YL, and XT collected and administered the serum. JK, XL, YL, and DM conducted the novel object and object place recognition tests. DW and MN performed HE staining. DW, YL, and MN performed immunostaining. DM, YL, and MN performed Western blotting. DW, JK, and XDL analyzed the data. DW and JK wrote the manuscript. LS, BL, and YZ modified the manuscript. All authors read and approved the final manuscript.

## Conflict of Interest

The authors declare that the research was conducted in the absence of any commercial or financial relationships that could be construed as a potential conflict of interest.

## Publisher's Note

All claims expressed in this article are solely those of the authors and do not necessarily represent those of their affiliated organizations, or those of the publisher, the editors and the reviewers. Any product that may be evaluated in this article, or claim that may be made by its manufacturer, is not guaranteed or endorsed by the publisher.

## Abbreviations

Aβ: amyloid-β
AD: Alzheimer's disease
ANOVA: analysis of variance
APOE: apolipoprotein E
APP: amyloid-β precursor protein
Arg1: arginase-1
CNS: central nervous system
DG: dentate gyrus
HRP: horseradish peroxidase
IBA-1: ionized calcium-binding adapter molecule 1
IF: immunofluorescence
IHC: immunohistochemistry
IL: interleukin
iNOS: inducible nitric oxide synthase
NOR: novel object recognition
OPR: object place recognition
PBS: phosphate-buffered saline
PS1: presenilin 1
SPs: senile plaques
SEM: standard error of the mean
SYN: synaptophysin
TBI: traumatic brain injury.
